# Exceptions, Paradoxes, and Their Resolutions in Chemical Reactivity

**DOI:** 10.1021/acs.joc.4c02246

**Published:** 2024-11-07

**Authors:** Guanqi Qiu

**Affiliations:** Max-Planck-Institut für Kohlenforschung, Kaiser-Wilhelm-Platz 1, 45470, Mülheim an der Ruhr, Germany

## Abstract

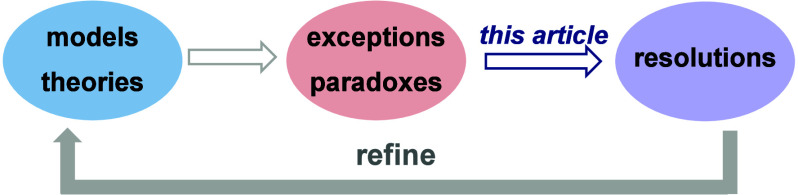

Progress in chemistry has primarily been framed through inductive processes, leading to the frequent emergence of exceptions and unexpected reactivities. These anomalies—ranging from surprising reactivity trends and paradoxical understandings to unanticipated parameter influences and unexpected successes or failures in synthetic methods—offer valuable insights that can drive scientific discovery. While it is commonly accepted that such exceptions can drive progress, many have been passively accepted without being explored for opportunities. Although numerous chemists have addressed exceptions and refined chemical models and theories, employing a systematic framework for actively exploring and understanding the underlying causes of exceptions could resolve paradoxes in broader contexts and create a greater impact than treating anomalies as isolated occurrences. This perspective demonstrates a proactive epistemic approach to uncovering the opportunities presented by exceptions and promotes deliberate, thoughtful engagement with paradoxes and anomalies. While the examples primarily focus on physical organic chemistry, the concepts are broadly applicable across various fields in chemical science. The thinking framework presented here is neither exhaustive nor prescriptive, but it outlines one of many potentially possible ways to inspire further development in how these anomalies could be harnessed for advancement.

## Introduction

Chemists formulate theories and models to rationalize data and predict outcomes in new chemical systems. With prolonged immersion in these models and our intuition as experienced chemists, certain observed reactivities may appear “more expected” than others. Unexpected reactivities often arise, manifesting as exceptions and/or paradoxes. Advancements in chemistry have primarily been driven by inductive processes^1^ (although deductive reasoning often contributes in smaller, incremental steps). Therefore, exceptions in chemical theories are ubiquitous and indicate an incomplete understanding, suggesting that the chemical system is not accurately described (i.e., the “boundary” that decides whether a reaction is relevant to the theory is not well-defined) and/or that the theory has limitations. Therefore, viewing exceptions not as anomalies but as opportunities to deepen our understanding is crucial.

While the concept of scientific discovery described here is not new at the philosophical level,^2^ it can be more concretely contextualized in chemistry, where many exceptions and paradoxes have been passively accepted rather than actively scrutinized. For instance, in synthetic method studies, whether unsuccessful substrates should (or at least better) be reported in substrate scope investigations remains a topic of ongoing debate. These failed substrates, while often not the focus of the main studies, can reveal important insights into the limitations of current mechanistic models and how they might be refined. They suggest that the hypothesized reaction mechanisms may have been oversimplified or overgeneralized, and/or that the functional groups undergoing the key transformations may not dominantly represent the chemical properties of the substrates within the given reaction system. With a more widespread appreciation of the epistemic significance of how to systematically address exceptions, reporting unsuccessful substrates could eventually become standard practice rather than a topic of debate. While many chemists have been engaging with exceptions and refined chemical models and theories, adopting a systematic framework to actively investigate and understand the underlying causes of these exceptions could resolve paradoxes in broader contexts and have a greater impact than treating anomalies as isolated events. For example, we can identify a number of misconceptions, but what is the specific essence of each different misconception? Likewise, some widely accepted models are fundamentally incorrect but still useful. Why, for instance, is VSEPR (Valence Shell Electron Pair Repulsion) theory—despite lacking a solid physical basis—still widely used? Additionally, every approximation made in reactivity models involves a trade-off—how do we determine the most appropriate balance in each situation? Targeted, systematic scrutiny of unexpected reactivities can lead to asking essential, yet often overlooked, questions, ultimately opening pathways to address many long-standing problems.

**Scheme 1:**
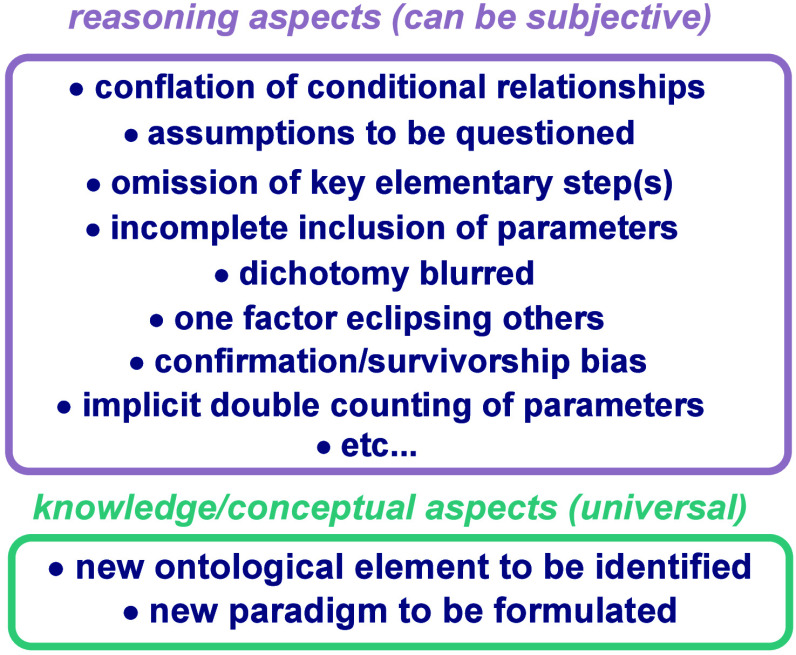
List of Possible Origins of Exceptions and Paradoxes

To this end, inspired from the logic of scientific discovery in general,^2,3^ I postulate some origins of unexpected chemical reactivities across different contexts, offering one (of potentially many) possible systematic approach to dealing with paradoxes and exceptions that may facilitate continuous improvement of models more broadly. While the emblematic examples focus on physical organic chemistry, the concepts discussed are universally applicable across different fields in chemical science. I have compiled a list of origins of exceptions and paradoxes (Scheme 1). Importantly, this list is certainly not exhaustive. I categorize these origins into two groups, with the first group focusing on reasoning. In an ideal realm of reasoning, these types of perceived paradoxes would be “nonexistent”, meaning they would not be considered paradoxical but rather different cases still within our framework of understanding. In reality, however, perfect reasoning is unattainable, making these types of perceived paradoxes very common. Given the diverse areas of expertise and thinking styles, the identification of something as paradoxical can be subjective; a result may seem paradoxical to some but not to others. Nonetheless, while these origins of exceptions and paradoxes may be interpreted subjectively at an individual level, their occurrence is statistically universal, reflecting broader patterns in chemical reasoning. The latter aspect, unavoidable even with perfect reasoning, arises when one or more key ontological elements are/were unidentified and cannot be replaced by combining known elements in new ways. This often paves the way for the formulation of a new paradigm. Consequently, these origins are not subjective but universally applicable. I select some origins of exceptions and paradoxes listed in Scheme 1 and provide some examples for each. *Importantly, while each example is chosen to illustrate one origin, some examples manifest more than one postulated origin. Additionally, these origins are not completely orthogonal to each other but can exhibit some overlap*. While some of the chosen examples might be controversial, I intend to use them to illustrate a way of thinking rather than to dogmatize the conclusions themselves.

### Origin 1. Influence and Determination Conflated

*While a factor may not fully determine an outcome, it can still play a significant role in influencing it. Even if a property is not entirely determined by a factor, that factor can still affect the property. This conflation between influence and determination often leads to misconceptions in chemical reasoning*.

**Figure 1:**
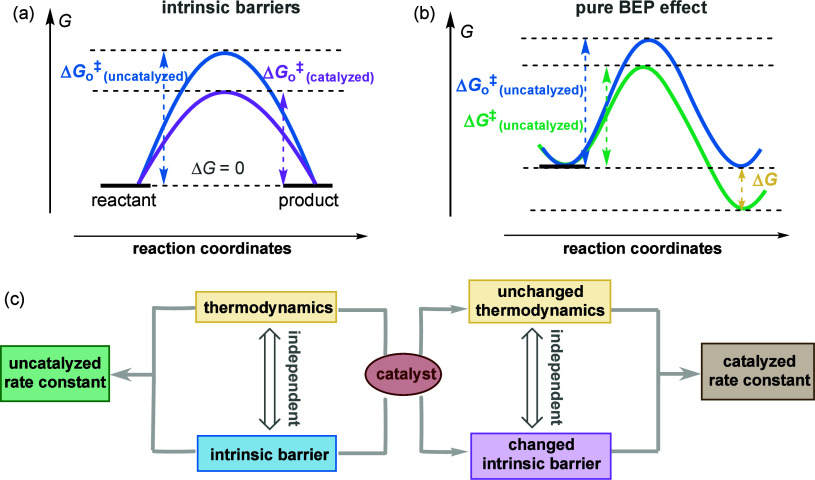
(a) Definition of intrinsic barrier and the comparison between the intrinsic barriers of uncatalyzed and catalyzed reactions. (b) The pure thermodynamic Bell–Evans–Polanyi (BEP) effect in uncatalyzed reactions. (c) Resolution of the interplay of thermodynamics, catalysts, intrinsic barriers, and kinetics (the colors are intentionally selected to illustrate the effect of mixing).

It is common to see many chemists having a misconception that activation free energy cannot depend on thermodynamic driving force. At the same time, the Bell–Evans–Polanyi (BEP) principle,^4,5^ which describes a linear correlation of reaction rate constants (or other activation parameters within the same family of reactions) with reaction free energy, is also well recognized. This principle relates qualitatively to the Hammond postulate,^6^ which posits that thermodynamic driving force does affect activation free energy. Unlike the examples chosen for other origins, which address limitations in chemical reactivity theories, this example demonstrates a paradox that emerges from cognitive dissonance and compartmentalized thinking in engaging with these reactivity theories. One possible reason for this paradoxical observation could be a common logical conflation between sole determination and partial influence. When we conflate determination and influence, we might oversimplify complex relationships, leading to the separation and isolation of ideas that should be integrated. This oversimplification can result in compartmentalized thinking, making it harder to recognize conceptual inconsistencies. For example, the well-known textbook principle that catalysts change the rate constant without altering the equilibrium constant can, when combined with the common conflation between sole determination and partial influence, inadvertently reinforce the misconception that thermodynamics does not affect kinetics. I attempt to demonstrate how resolving the conflation between sole determination and partial influence can clarify the misconception and ultimately address the aforementioned paradoxical cognitive observation through the use of Figure 1. Regarding the interplay between thermodynamics and kinetics, the Marcus model dissects reactivity into thermodynamic-dependent and thermodynamic-independent components. It expresses the free energy of activation Δ*G*^⧧^ by a combination of the reaction’s free energy Δ*G* and the intrinsic barrier Δ*G*_0_^⧧^, which corresponds to Δ*G*^⧧^ for Δ*G* = 0 (Figure 1a), in the linear approximation (Δ*G*^⧧^ = Δ*G*_0_^⧧^ + αΔ*G*) known as the Leffler equation.^7^ The intrinsic barrier is considered to reflect the reorganization energy required for the deformation of the nuclear coordinates of the reactant state to that of the product state, which represents the inherent nature of each family of reactions independent of the thermodynamic driving force.^8^ The Brønsted slope α indicates the sensitivity of the reaction rates to changes in driving force. Both Δ*G*_0_^⧧^ and α are intrinsic properties of each family of reactions.^9,10^ For constant Δ*G*_0_^⧧^ and α, the Leffler equation is reduced to the BEP principle (Figure 1b), which also means that between different intrinsic reactivities, the Hammond postulate is no longer relevant. While catalysts do not change the thermodynamic driving force, they change the rate constant by changing the intrinsic barrier (Figure 1a). Figure 1c assembles all of these ideas; thermodynamics does not affect the intrinsic barrier, but affects the thermodynamic contribution to the reaction barrier as per BEP principle, thus affecting the resultant rate constants; the difference in rate constant between the catalyzed and the uncatalyzed reactions is attributed to the changes in intrinsic barrier that the catalyst caused; thermodynamics and intrinsic barrier do not depend on each other, but the observed rate constant depends on both. In this holistic picture, both thermodynamics and the catalyst *influence* the kinetics, but neither solely *determines* it.

**Scheme 2:**
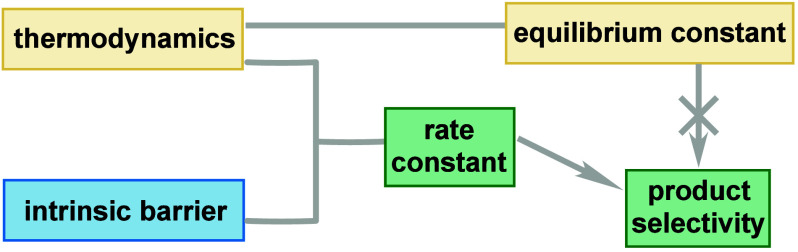
Role of Thermodynamics in Reactions under Kinetic Control

Another example, also related to the misconception that thermodynamics does not affect kinetics and demonstrating the conflation between sole determination and partial influence, involves the misinterpretation of the concept of kinetic versus thermodynamic control. This concept states that for irreversible reactions, product selectivity is determined by the kinetic barrier rather than the equilibrium constant. However, even though thermodynamic equilibrium is not reached in reactions under kinetic control (i.e., the equilibrium constant is irrelevant in this context), the thermodynamic driving force can alter the kinetic barrier according to the BEP principle, thus affecting the rate constant (Scheme 2) and, by extension, the product selectivity for reactions under kinetic control. That is, thermodynamic equilibrium *does not determine* the kinetics, but still *influences* the kinetics. We can also consider the principle of microscopic reversibility, which asserts that the forward and reverse rates of each elementary step are equal at equilibrium.^11^ This principle directly connects thermodynamics and kinetics (i.e., influence). However, thermodynamics does not solely determine kinetics, as another factor, the intrinsic barrier, equally influences both the forward and reverse rate constants. By discerning the distinctions between sole determination and partial influence, the misconception that activation free energy cannot depend on thermodynamic driving force can be clarified, thereby realigning it with the understanding of the BEP principle. Conflation between determination and influence, along with other logical fallacies (such as affirming the consequent, denying the antecedent, confirmation bias, and circular reasoning), is responsible for a significant number of incorrect ad hoc mechanistic determinations. I anticipate that clearer distinctions between determination and influence, along with addressing these other logical fallacies, will help dispel broader misconceptions and enhance cognitive resonance across many other areas of chemistry as well.

### Origin 2. Assumptions to Be Questioned

*Many theories and rules regarding chemical reactivity are built on foundational assumptions. When these assumptions no longer hold true under certain conditions, the associated rules can break down*.

**Figure 2:**
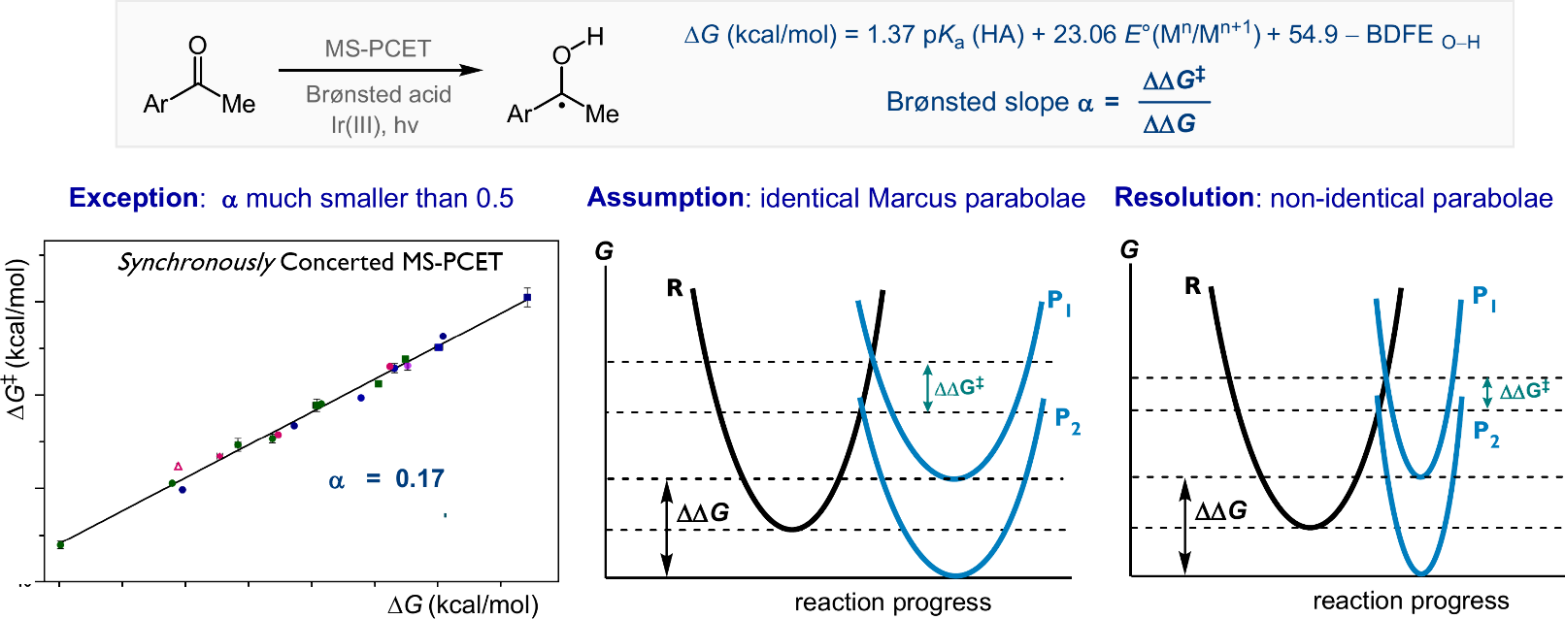
Illustration of the importance of evaluating underlying assumptions. The plot was reproduced with permission from ref (12). Copyright 2019 ACS Publications.

I select a system of multisite proton-coupled electron transfer (MS-PCET) as the illustrating example in this case (Figure 2).^12^ MS-PCET leads to a net transfer of hydrogen atom, with the electron and the proton exchange with different sites.^13^ In this system, an aryl ketone establishes a hydrogen-bonding pre-equilibrium adduct with a Brønsted acid, where the bound complex undergoes PCET upon excitation of a photoreductant, forming a ketyl radical intermediate. The driving force is jointly determined by the p*K*_a_ of the acid and the redox potential of the photoreductant.^14−16^ A strong linear correlation between the rate constant and the driving force is obtained no matter which component(s) of the driving force (i.e., acid or photoreductant) is varied. Mechanistically, it strongly suggests that the PCET reduction of aryl ketones is synchronously concerted, that proton and electron transfer have progressed to the same degree at the transition state.^17,18^ However, the correlation constant, Brønsted slope α, is much smaller than the value of α = 0.5 predicted by classical Marcus theory,^19^ meaning a much less sensitive rate-driving force dependence.

The underlying assumption for the expected α = 0.5 is that the Marcus parabolae of the reactant and the product states have identical shape and differ only in their vertical displacement, which represents the driving force. The Marcus Parabola describes the energy of a collection of atoms in terms of the nuclear position, which is a function of numerous factors such as bonding, conjugation, sterics, solvation, and noncovalent interactions. Each of these affects the parabola’s shape. By comparing the reactant and product states in reductive PCET, it is evident that the charge distribution, the hydrogen-bonding framework, and solvation have all changed. Thus, the shapes of the parabolae are very likely to be different, invalidating the assumption of identical-parabolae.^20−22^ Note that while the Marcus parabola representation is practically very useful, it is a very simplistic reduction of the N-dimensional potential energy surface.^19^ All models could have the problem of oversimplification (*vide infra: “useful” versus “correct”*). A more physically intuitive explanation is the principle of nonperfect synchronization: the factors determining the energy of the product have developed to different degrees compared to the primary bond changes, influencing the responsiveness of the reaction barrier to the change in driving force.^23^

There are many other examples concerning the validity of assumptions. For instance, in the photochemistry of electronically excited states, the Kasha rule dictates that excited states always emit from the lowest energy level of a given spin multiplicity, which assumes that the internal conversion is faster than emission.^24^ However, anti-Kasha emission happens when internal conversion occurs less rapidly than emission, usually as a result of large energy gaps between excited states.^25^ In a separate example, ligand field theory assumes that in organometallic complexes, ligands are more electronegative than metals and have frontier orbitals of lower energy than those of the d orbitals of electropositive metals.^26^ When moving to the right in the d-block and approaching the transition-metal/main group boundary, the d orbitals become more core-like, making their cations more electronegative, eventually arriving at a point where they are lower in energy than the ligand frontier orbitals, resulting in inverted ligand field theory.^27^ These examples should remind us to examine these two aspects of each scientific conclusion: 1) given that the assumptions are valid, whether the deduction is logically correct; and 2) whether the assumptions themselves are valid. Asking more “what if” questions should always be beneficial in critically evaluating assumptions.^28,29^

Incomplete consideration of factors is another common cause for exceptions and paradoxes. In the next two origins, I discuss two key facets of factor inclusion that could be expanded.

### Origin 3. Omission of Concurrent Path(s)

*The observed reactivity may contradict what is predicted from one key path. Upon considering side path(s), the overall conclusion is no longer paradoxical*.

**Figure 3:**
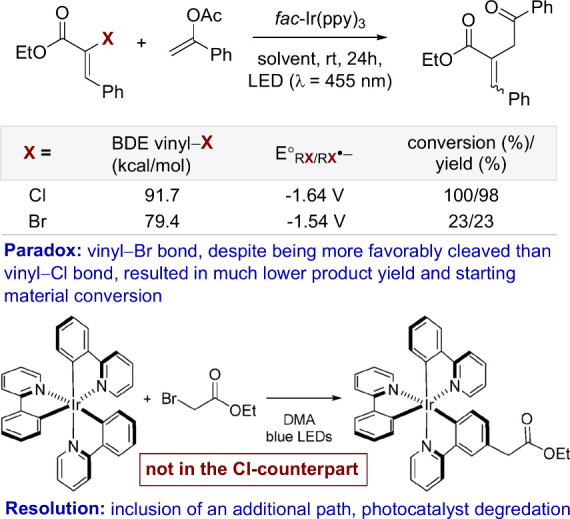
Illustration of how the omission of concurrent path(s) can cause paradoxical reactivity trends.

Here, the illustrative example is a study of visible-light-mediated activation of vinyl chlorides derived from α-chloro ethyl cinnamates via oxidative quenching of excited photocatalyst *fac*-Ir(ppy)_3_ (Figure 3).^30^ Upon photoelectron transfer and chloride extrusion, the corresponding vinyl radical can be efficiently trapped by enol acetates, giving rise to 1,4-dicarbonyl compounds. In agreement with reduction potentials and bond dissociation energies (BDEs), the α-fluoro cinnamate substrate, which has the strongest vinyl–X bond (BDE = 123.7 kcal/mol), does not allow for the formation of a vinyl radical. However, the trend between the α-chloro cinnamate and the α-bromo cinnamate substrates is paradoxical under this mode of analysis. The data presented indicate that the C_vinyl_–Cl bond is both significantly stronger and more challenging to be reduced than the corresponding C_vinyl_–Br bond. Nevertheless, under the optimized conditions, the α-bromo cinnamate substrate resulted in a significantly lower product yield. The resolution to this paradox comes from taking another concurrent event into consideration: photocatalyst degradation. Ethyl bromoacetate, formed as a byproduct upon the reaction of the vinyl bromide with the enol acetates, acts as an efficient catalyst poison for *fac*-Ir(ppy)_3_ due to acid-induced ppy ligand radical functionalization. In contrast, ethyl chloroacetate, formed in the reaction of the α-chloro cinnamate substrate, has not been found to have this effect. Consequently, in the case of the α-bromo substrate, the productive reaction halted at low conversion due to deactivation of the photocatalyst.^31,32^

### Origin 4. Incomplete Inclusion of Molecular Factors

*One molecular property might suggest an outcome opposite to what is observed, but the omitted molecular property could favor an alternative result. Upon considering broader molecular properties, the outcome is no longer paradoxical*.

**Figure 4:**
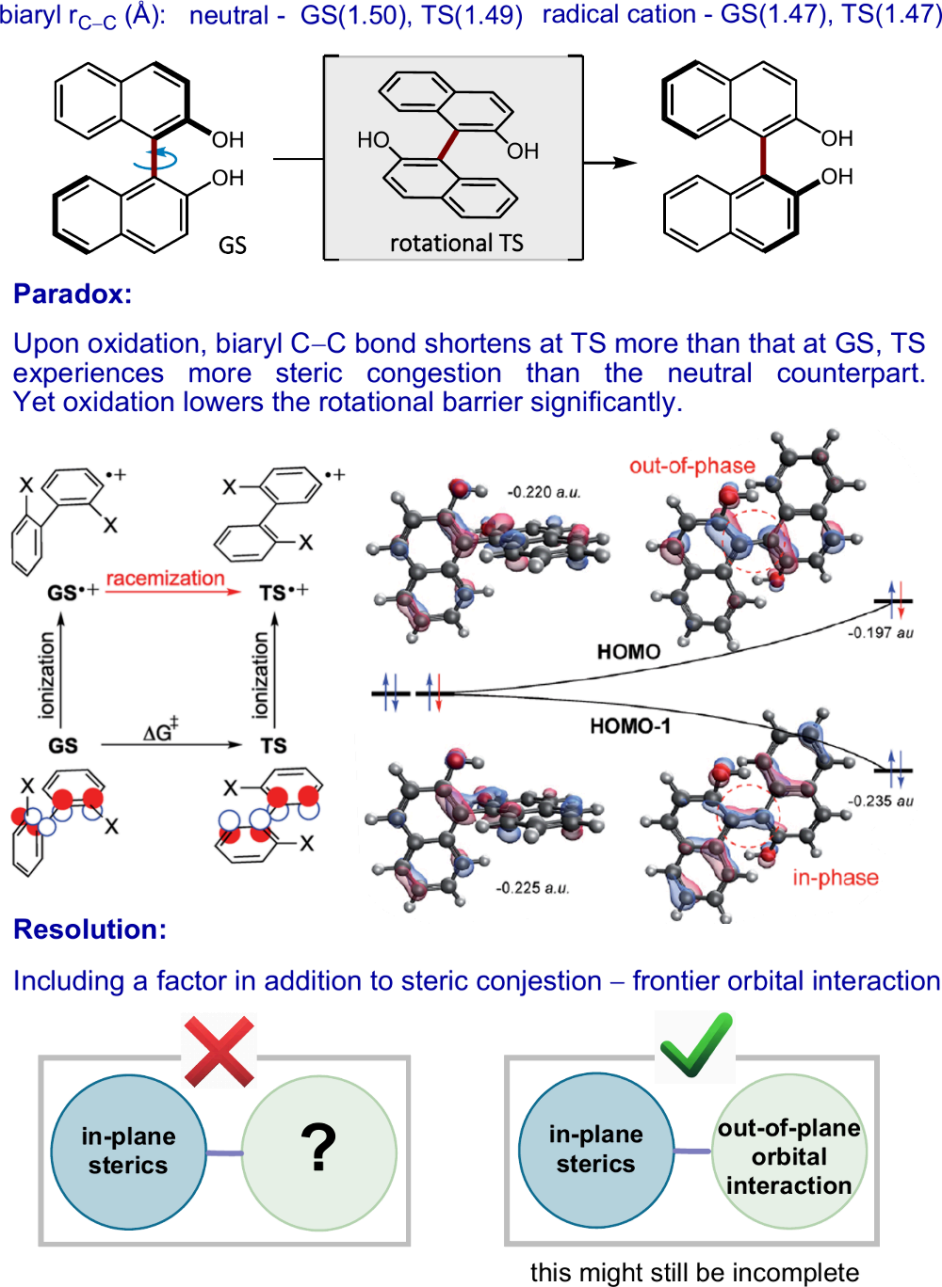
Illustration of how incomplete inclusion of parameters causes unexpected results. The contextual figures were reproduced with permission from ref (33). Available under a Creative Commons Attribution 3.0 Unported License. Copyright 2019 RSC Publications.

An illustrative example comes from the study of the rotational barriers of biaryl compounds (Figure 4).^33^ At first glance, single electron oxidation appears to contradict expectations; it shortens the biaryl C–C bond, seemingly increasing steric congestion at the transition state compared to the neutral counterpart, without involving dispersion forces. Paradoxically, despite this bond contraction, the rotational barrier decreases significantly upon oxidation. This paradox resolves upon considering an additional factor besides bond length: the frontier orbitals. Initially, focusing on these orbitals might seem counterintuitive, especially in a context devoid of bond formation. However, removing an electron from the HOMO stabilizes the planar transition state to the point that it becomes an intermediate on the potential energy surface. This stabilization, alongside the bond shortening in radical cations—particularly noticeable in the rotational transition state and achiral minimum—is due to enhanced bonding character between the carbon atoms. Arene radical cations’ greater preference for planarity relative to their neutral counterparts is another outcome of this stabilization, driven by the net bonding interaction between the naphthalene π-systems when the HOMO is partially unoccupied. In essence, while steric hindrance alone would suggest an increase in the rotational barrier, the inclusion of frontier orbital interactions offers a consistent explanation.

In general, although the inclusion of the additional factor appears to make the data no longer paradoxical, the analysis may still not fully encapsulate the complexity of a phenomenon. Other underlying factors might be overshadowed by the focused study of the two parameters for one specific molecule. In structurally similar molecules, different yet unstudied parameters could prove significant, suggesting a need for a broader, albeit still incomplete, understanding.

### Origin 5. Synergistic Effects Neglected

*When two or more factors play important roles, the net effects may not be additive, but rather synergistic. Such synergistic effects could lead to results that are paradoxical to the factors operating additively*.

**Figure 5:**
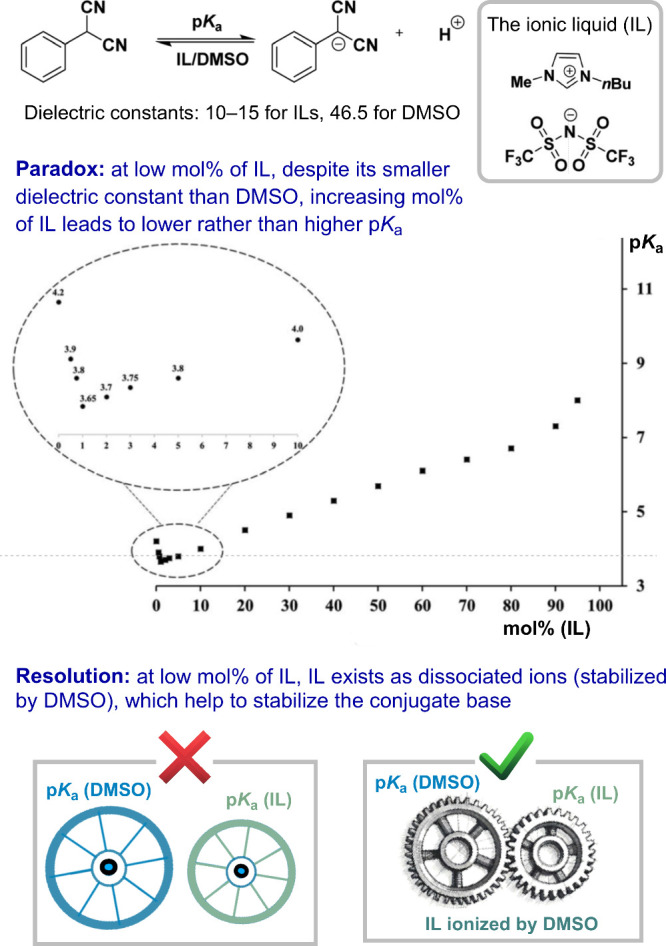
Illustration of how neglecting synergistic effects causes paradoxical results. The plot was reproduced with permission from ref (34). Available under a Creative Commons Attribution 3.0 Unported License. Copyright 2020 RSC Publications.

Figure 5 illustrates this idea in a study of the solvent effects on the p*K*_a_ of a C(sp^3^)–H bond.^34^ The dielectric constants of a typical ionic liquid (IL) and DMSO differ significantly,^35−37^ and these solvents are readily miscible. If the IL and DMSO behaved additively, one would expect a linear relationship between the p*K*_a_ of the compound and the ratio of IL to DMSO; specifically, a higher percentage of IL should correlate with a higher p*K*_a_. This is indeed observed at moderate and high percentages of IL. However, at low percentages of IL, the opposite trend occurs. The paradox is resolved by a synergistic effect between IL and DMSO. At moderate and high concentrations of IL, IL exists as tightly bound neutral ion pairs. At lower concentrations, however, IL exists as dissociated ions, solvated by DMSO. The dissociated ions stabilize the conjugate base, thereby increasing acidity.^34^ This example highlights the importance of considering synergistic effects where IL and DMSO interact in a nonpurely additive manner, demonstrating mutual interactions.

Additionally, synergistic effects manifest in multicatalyst reaction optimization campaigns. A traditional approach is optimizing each component individually then combining the best-performing catalysts from the individual optimization studies. However, this approach does not always lead to the best overall performance if there is a negative synergy between different components of catalysts. These interactions can detract from the efficacy expected from the optimal performance of each catalyst in isolation, highlighting the need for holistic optimization approaches that consider the interplay between catalysts to truly enhance the desired reaction.

### Origin 6. Dichotomy Blurred

*The perceived paradox in some cases arises from our longstanding expectation of a clear division between two classes of bonding or mechanistic scenarios. However, this division may not be as pronounced as we assume. Instead, it could span a spectrum of behaviors depending on the relative influences of various parameters. Rather than attributing behavior to a single factor, it is necessary to recognize that multiple factors contribute to the net outcome, resulting in a continuous spectrum of potential outcomes*.

**Figure 6:**
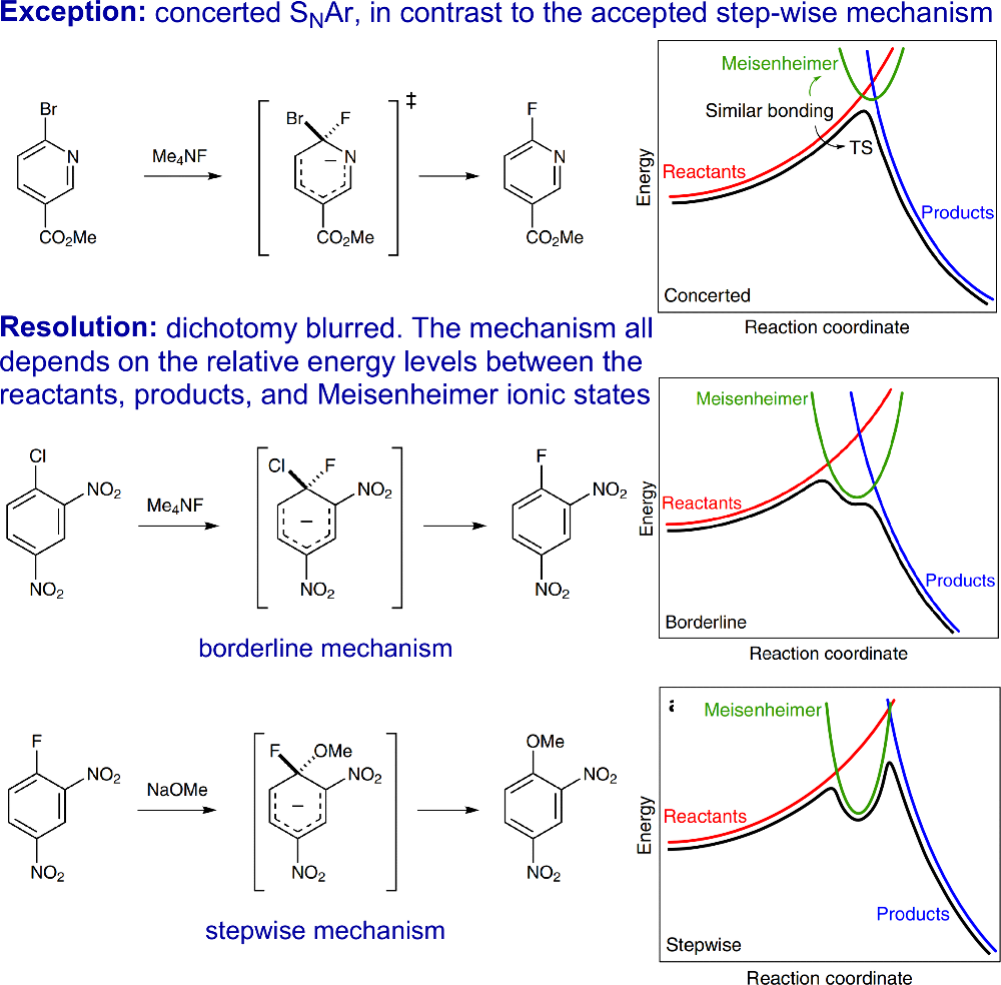
Illustration of blurring the dichotomy in mechanistic situations of nucleophilic aromatic substitution (S_N_Ar) reactions. The figure was reproduced with permission from ref (41). Copyright 2018 Nature Portfolio Publications.

In textbooks, nucleophilic aromatic substitution (S_N_Ar) reactions are generally explained to proceed through a two-step addition–elimination sequence via a discrete, nonaromatic Meisenheimer complex. This intermediate is usually stabilized by electron-withdrawing groups (EWGs), leading to a minimum along the reaction coordinate.^38,39^ Recently, however, an increasing number of concerted nucleophilic aromatic substitutions have been reported, featuring better leaving groups and less stabilizing EWGs.^40−42^ These findings are supported by, for example, a study involving qualitative Marcus theory (Figure 6),^41^ which suggests that the reaction pathway depends critically on the energy of the Meisenheimer complex relative to the “potential energy surfaces” (in the language of the valence bond theory in modeling reactivity) of the starting materials and products.^43−45^ When this complex is lower in energy, the reaction proceeds stepwise; if it is higher, the mechanism is concerted, with the transition state becoming the minimum of the Meisenheimer curve. Borderline cases, such as when a stabilized anion is adjacent to a good leaving group, show that the Meisenheimer complex may merely form a shallow minimum or shoulder on the reaction coordinate, indicative of the blurred lines between mechanisms. In reactions consistent with concerted S_N_Ar, the transition state structures were found to possess characteristics of a Meisenheimer intermediate rather than those of the aromatic starting material.

When the Meisenheimer structure is less stable, reactions tend to be concerted. If this structure is highly stabilized but leaving group elimination is facile, a borderline situation arises, underscoring that the distinction between concerted and stepwise mechanisms is not dichotomous but depends on the relative energy levels between the reactants, products, and Meisenheimer ionic states. In some instances, the intermediate could behave more like a transition state. This continuum is further explored in another study that surveyed various substituents and nucleophiles, mapping out the range between concerted and stepwise mechanisms.^46^ A concerted mechanism can be synchronously or asynchronously concerted, with varying degrees of asynchrony; when it becomes highly asynchronous, it transitions to a stepwise mechanism.^47^ With the idea of a blurred dichotomy, many mechanistic exceptions should no longer be surprising.

What we can also learn is that almost no class of reactions is inherently meant to be either concerted or stepwise; they only happen to exhibit one mechanism or another for most of the cases we have encountered. The prevailing beliefs regarding which class of reactions corresponds to which mechanism are typically based on the most commonly encountered examples. The collection of these examples often has led to a statistical, but not directly causal, association with a particular mechanism. This predominant belief that a certain class of reactions must follow a specific mechanism may illustrate another origin—*survivorship bias*.

### Origin 7. New Ontological Element(s) to Be Identified

*This origin diverges from the reasoning aspects discussed above, focusing instead on the necessity for new conceptual frameworks. The introduction of new ontological elements becomes essential when the current entities of reactivity and mechanism are insufficient. This typically occurs in situations where anomalies reveal fundamental gaps in our understanding that cannot be bridged by merely adjusting existing concepts*.

**Figure 7:**
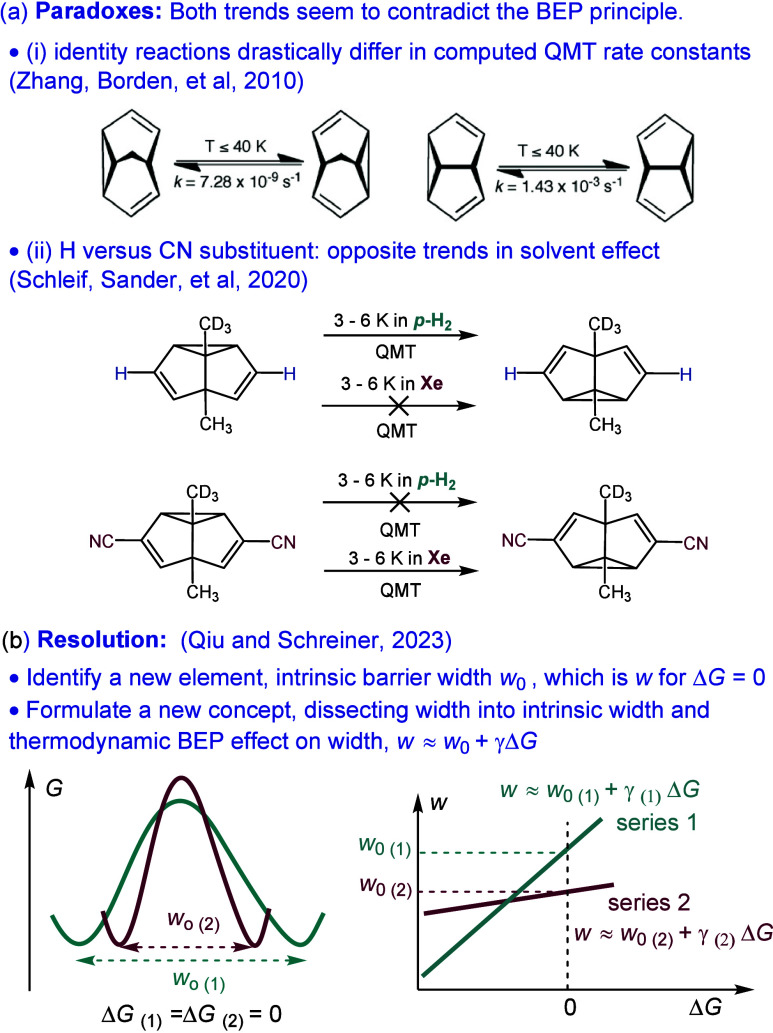
Examples illustrating the possible need/benefit to identify new elements and formulate new paradigms in resolving paradoxical reactivity trends. (a) Paradoxical reactivity trends in quantum mechanical tunneling. (b) Resolving the paradoxes by identifying a new element and formulating a new concept. For simplicity, zero-point vibrational energy levels are omitted. Note that the new concept is the *dissection* of the actual barrier width into the *intrinsic* and the *thermodynamic* (BEP effect) components, not the significance of the actual barrier width itself.

An illustrative example can be found in studies of quantum mechanical tunneling (QMT) reactivity, where conflicting trends in QMT behavior prompted the proposal of a new conceptual framework. QMT, a consequence of the wave nature of particles, implies that a particle can pass through a potential energy barrier without having sufficient energy to overcome it, and has recently been more broadly taken seriously in chemical reactions.^48−54^ QMT reactivity is highly sensitive to the width of the reaction energy barrier. Many studies have reported that while reaction barrier width (readout by QMT reactivity) is strongly influenced by the thermodynamic driving force, substituents, and matrix environments, the observed trends often conflict.^55−59^Figure 7a illustrates two examples where QMT is responsible for extremely high percentages of the overall reactivity, showcasing conflicting trends in QMT reactivity. In Figure 7a(i), both reactions are identity reactions with zero driving force, yet the computed QMT rate constants differ drastically.^58^ In Figure 7a(ii), the H-substituted compound exhibits significantly more reactive QMT than the CN-substituted compound in a *para*-H_2_ matrix, whereas the results are reversed in a xenon matrix.^55^ Both of these QMT reactivity trends seem to contradict the BEP principle. Noticing that while the Marcus dissection of the reaction barrier height into thermodynamically independent (intrinsic barrier) and dependent (BEP) contributions models the interplay of reaction rate and driving force effectively, a similar concept for barrier width was absent. With that, the intrinsic barrier width (*w*_0_) was defined as the barrier width at zero thermodynamic driving force.^60^ Analogous to intrinsic barrier heights (that reflect reorganization energy), intrinsic barrier width reflects the reorganization of nuclear coordinates at zero thermodynamic driving force inherent to each family of reactions. As a result, the *actual* barrier widths of the two identity reactions in Figure 7a(i) differ because their *intrinsic* barrier widths are different, which is consistent with their considerable differences in geometric rearrangement. This led to the conceptualization of the barrier-width counterpart to the Leffler equation (Δ*G*^⧧^ = Δ*G*_0_^⧧^ + αΔ*G*, *vide supra*), where the barrier width can also be dissected into an intrinsic component that is thermodynamic-independent and a thermodynamic component as per the BEP effect: *w* = *w*_0_ + γΔ*G*, where γ is analogous to the Brønsted slope, indicating the sensitivity of barrier width to thermodynamic changes.^60^ With this new concept, we might be able to rationalize the conflicting trend in Figure 7a(ii). Reactions in the two different solvents (*para*-H_2_ or xenon) perhaps belong to different sets of rate-driving force correlation lines, with different intrinsic barrier width *w*_0_ and sensitivity of width versus driving force (γ). One has smaller barrier width at low driving forces, while the other has smaller barrier width at high driving forces (Figure 7b). Solvation effect has been found to play important roles in influencing Δ*G*_0_^⧧^ and α,^23^ and they are likely also important in influencing *w*_0_ and γ. While this new concept of deconvoluting barrier width would not resolve all the conflicting trends in reactions where barrier widths are important (and is certainly not the sole resolution), it at least offers a reasonable hypothesis and meaningful guidelines for future endeavors.

After discussing all the postulated origins above, the next question is when to identify and introduce new elements into reactivity models. It is essential to avoid creating terms that are redundant, which would only complicate matters unnecessarily. A practical rule of thumb might be to consider defining new elements when the features cannot be reproduced by taking quantitative, linear combinations of formerly defined elements. For example, ionic or covalent bonding are not a dichotomy but are rather two ends of a spectrum. However, in some cases, such as with F–F and Me_3_Si–Cl, experimental results contradict the models of both ionic and covalent bonding and cannot be rationalized merely as “partially ionic plus partially covalent”. This discrepancy led to the proposal of a new type of bonding known as charge-shift bonding.^61−64^ The concept of the charge-shift bonding is not without controversy, highlighting once again the necessity of continuously questioning the existing paradigm. Another example concerns dynamical theories of reaction product selectivity, which were invoked when transition-state theory fails to adequately explain the experimentally observed product selectivity.^65−68^ It is crucial to consider dynamic reactivity in these scenarios, while also avoiding the premature attribution of discrepancies to dynamic effects without thorough investigation.^69^

Importantly, the two classes of exceptions—imperfect reasoning (Origins 1–6) and insufficient conceptual elements (Origin 7)—do not form a sharp dichotomy but can be intertwined. Therefore, rather than separating these classes, the goal is to deconvolute them and understand their conceptual differences. For instance, in Origins 3 and 4, the omitted path or parameter might be something already known or something yet to be identified. The situation where “this exception can be resolved only if we identify new elements” or “existing elements would suffice, we just have not picked the right ones” is not always clear. This leads to a practical framework: first, assess whether including all the known paths and parameters can resolve the paradox; if not, consider proposing new elementary steps or identifying new parameters. This ambiguity underscores the importance of understanding their differences. The key is to remain intellectually open-minded. We can make a greater effort to find the known elements that fit the data while remaining receptive to the possibility of new elements in the future. We can also propose new elements but consider them “in probation” rather than as settled conclusions.

#### Useful versus Correct

**Figure 8:**
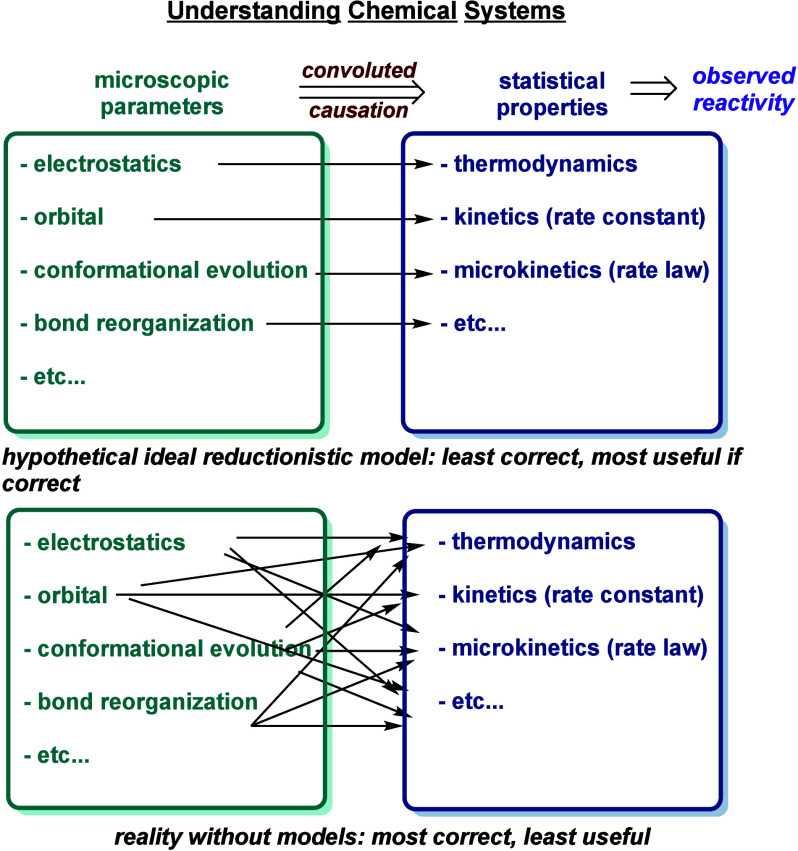
Two extreme ends of the trade-off between “useful” and “correct” in chemical reactivity models.

In summary, exceptions and paradoxes are inherent to reactivity theories and models due to the complex interplay of multiple factors (Figure 8). The influence of any single property on chemical reactivity is intertwined with nearly all other properties of the molecules or their environment, though the degree of influence can vary greatly—some properties have a strong influence, while others are essentially negligible. The specificity (and its qualifications) directly affect how correct and how broadly useful a statement is. For example, a general statement “a photocatalyst can degrade during photocatalytic reactions” is completely correct, as it is supported by numerous examples of photocatalyst decomposition. In fact, just one piece of evidence is sufficient to make this statement correct, reducing its value in helping us understand other photocatalytic reactions. In contrast, a more specific statement such as “the presence of a Lewis acid is likely to cause photocatalyst degradation” might be incorrect, but it is far more useful because it offers a clearer direction for investigation. Theories and models strive to find an optimal balance. Therefore, the trade-off between “useful” and “correct” is inevitable, making exceptions and paradoxes prevalent. As mentioned earlier, reporting unsuccessful cases in synthetic method papers helps probe the boundaries of existing mechanistic understanding. This does not imply an obligation to change current models immediately. While we strive to make mechanistic models more rigorous, increasing complexity can reduce generality. If each reaction requires a distinct mechanism, it becomes akin to having no mechanistic model at all. The most important aspect is understanding this trade-off and adjust our position on this spectrum based on the specific goals of each situation. Another example of this trade-off can be seen in the models used to describe chemical structures.^70^ Identifying new elements and formulating new paradigms can fundamentally alter the “equilibrium constant”, enabling the development of theories and models that are both more correct and more useful.

## Conclusions

In the end, simple models are the language in which we understand chemistry. For the models to remain straightforward, exceptions and paradoxes are then inevitable. I have explicitly shown how unexpected behaviors often reveal limitations and gaps within these models, serving not as obstacles but as crucial indicators guiding us toward deeper insights. Such logic of scientific discovery, while long established at the philosophical level, remains under-contextualized in the field of chemistry. Often, obvious aspects can be overlooked for many years. I have presented a systematic framework to positively engage with these anomalies, as an attempt to reduce the oversight of obvious aspects. The origins of exceptions and paradoxes—from imperfect reasoning to the need to formulate new concept—underscore the need to challenge our presumptions and open to new paradigm. The categories discussed here are not exhaustive but represent one possible pathway to frame these phenomena, aiming to spark thoughtful discussion and promote intellectual open-mindedness. After my attempt, and others like it, even though our understanding would likely remain as clouded as before, the progress made by advancing from nascent awareness to informed bemusement ought not to be underestimated. Instead of saying ‘the more we know, the less we know’, I would offer a more positive and active rephrasing, ‘the more we know, the more we know *what* we do not know’, and thus, opportunities arise to learn even more. Increasing the intellectual sensitivity to seize such “opportunities” is my fundamental incentive for writing this article.

## Data Availability

The data underlying this study are available in the published article.

## Notes

The preprint of this work is available on ChemRxiv: Qiu, G. *Exceptions, Paradoxes, and Their Resolutions in Chemical Reactivities*. ChemRxiv, 2024. DOI: 10.26434/chemrxiv-2024-wp0vg.
